# Comprehensive Profiling of Free Amino Acids in Litchi (*Litchi chinensis* Sonn.) Germplasm and Their Implications for Flavor Quality

**DOI:** 10.3390/foods14234051

**Published:** 2025-11-26

**Authors:** Yingjie Wen, Fachao Shi, Yonghua Jiang, Hailun Liu, Qian Yan

**Affiliations:** 1 Key Laboratory of South Subtropical Fruit Biology and Genetic Resource Utilization, Ministry of Agriculture and Rural Affairs, Institute of Fruit Tree Research, Guangdong Academy of Agricultural Sciences, Guangzhou 510640, China; wenyingjie@gdaas.cn (Y.W.); shifachao@gdaas.cn (F.S.); jiangyonghua@gdaas.cn (Y.J.); liuhailun@gdaas.cn (H.L.); 2 Guangdong Provincial Key Laboratory of Science and Technology Research on Fruit Trees, Institute of Fruit Tree Research, Guangdong Academy of Agricultural Sciences, Guangzhou 510640, China

**Keywords:** *Litchi chinensis*, free amino acids, germplasm resources, flavor quality, hierarchical cluster analysis

## Abstract

*Litchi chinensis* Sonn. is an economically and culturally significant fruit crop in China, valued for its distinctive flavor, which arises from the combined contributions of taste and aroma metabolites. While the accumulation of sugars, organic acids, and volatile terpenes in litchi has been extensively studied, the role of nitrogenous flavor compounds, particularly free amino acids (FAAs), remains poorly characterized across diverse germplasm. To address this gap, high-performance liquid chromatography (HPLC) was employed to quantify 20 free amino acids in the pulp of 148 distinct litchi germplasm accessions. Comprehensive statistical analyses, including non-parametric tests, correlation analysis, and hierarchical clustering, were performed to elucidate compositional variations. Alanine (Ala), glutamate (Glu), and γ-aminobutyric acid (GABA) were the most abundant FAAs, contributing strongly to sweetness and umami. FAA profiles differed significantly among genomic groups, and clustering analysis identified three major chemotypes: Glu-accumulating, GABA-accumulating, and Ala-accumulating types. This study provides the first large-scale survey of FAA diversity in litchi germplasm and establishes a foundation for selecting cultivars with desirable flavor attributes and for future genomic dissection of amino acid metabolism.

## 1. Introduction

*Litchi chinensis* Sonn. is a tropical and subtropical evergreen tree of the Sapindaceae family, native to southern China [1]. The fruit is highly esteemed for its sweet, juicy pulp and distinctive flavor [2,3]. As the primary center of origin for litchi, China has a cultivation history exceeding 2000 years, resulting in a rich diversity of germplasm resources [1]. The National Litchi and Banana Germplasm Repository conserves over 600 litchi accessions, including major cultivated varieties and wild relatives from around the world [4]. Long-term evolution and domestication have led to significant divergence in quality traits among cultivars [5]. Therefore, characterizing the quality attributes of different cultivars is crucial for the effective utilization of litchi germplasm and the development of innovative breeding strategies.

Flavor is a critical quality attribute of fruit, significantly influencing not only palatability and nutrient but also consumer purchasing decisions [6]. It is a composite perception resulting from the interaction of taste and aroma [7]. Taste is primarily determined by non-volatile compounds sensed in the oral cavity, including sugars, organic acids, and nitrogenous substances such as amino acids and nucleotides [7]. Aroma, on the other hand, is derived from the nasal perception of volatile organic compounds (VOCs), such as alcohols, aldehydes, acids, esters, and terpenes [8]. Changes in the composition or concentration of these flavor components can directly affect the overall sensory profile and quality of the fruit [9]. While current research on litchi flavor has extensively documented soluble solids, sugars, organic acids, or volatile terpenes [10,11], the role of nitrogenous constituents, particularly amino acids, has received comparatively less attention.

Amino acids are organic compounds containing both amino and carboxyl functional groups and exist primarily in bound and free forms [12]. Bound amino acids are not easily hydrolyzed during consumption and therefore have little immediate effect on flavor [13]. Conversely, free amino acids (FAAs) act as precursors for the synthesis of volatile compounds (e.g., alcohols, aldehydes, and esters) and bioactive components such as flavonoids, while also directly contributing to the flavor profile of foods [14,15]. In 1908, Kikunae Ikeda identified umami, elicited by L-glutamic acid, as the fifth basic taste modality alongside sweetness, saltiness, bitterness, and sourness [16]. Subsequent studies have revealed that different amino acids can elicit not only umami but also the other four basic tastes [17]. Therefore, analyzing the composition of free amino acids in litchi fruit is essential for understanding its nutritional value and flavor characteristics.

Although several recent studies have examined FAAs in litchi [3,18], these analyses typically focused on only a small number of cultivars or assessed FAAs as part of broader metabolomic screens without in-depth functional interpretation in relation to genetic diversity. To address these limitations, we performed a large-scale FAA profiling of 148 litchi germplasm accessions using high-performance liquid chromatography (HPLC). The resulting dataset not only allowed for the comprehensive characterization of FAA distribution patterns but also enabled the integration of phenotypic data with genomic groupings and sugar accumulation types, thereby advancing from mere description to functional insight. Furthermore, by applying cluster analysis, we successfully classified distinct FAA chemotypes, thereby providing a functional framework for germplasm utilization. This work identifies accessions with extreme FAA content for breeding and establishes a robust foundation for subsequent genome-wide association studies (GWAS) to uncover key genes governing amino acid metabolism in litchi.

## 2. Materials and Methods

### 2.1. Plant Materials

A total of 148 litchi germplasm accessions were obtained from the National Litchi and Banana Germplasm Repository (Guangzhou, China) for this study. The experimental orchard is located on a gentle slope, and all sampled trees were over 20 years old. Uniform soil fertility and consistent horticultural management were maintained throughout the orchard to ensure stable annual flowering and fruiting [5].

Fruit samples were harvested in the summer of 2022 at approximately 80% maturity, as determined primarily by pericarp color. For each accession, three individual trees were selected as biological replicates. From each tree, ten fruits of uniform size and color, free from pests, disease, or mechanical damage, were randomly collected. The fresh weight of the arils was recorded immediately after seed removal. The arils were then separated, flash-frozen in liquid nitrogen, and stored at −80 °C until further analysis.

### 2.2. Chemical Reagents and Standards

Seventeen amino acid standard stock solutions were obtained from Sigma-Aldrich (St. Louis, MO, USA), including alanine (Ala), arginine (Arg), aspartic acid (Asp), cysteine (Cys), glutamic acid (Glu), glycine (Gly), histidine (His), isoleucine (Ile), leucine (Leu), lysine (Lys), methionine (Met), phenylalanine (Phe), proline (Pro), serine (Ser), threonine (Thr), tyrosine (Tyr), and valine (Val). The concentration of Cys was 1.25 mmol/L, while all other amino acids were 2.5 mmol/L. Additional standards for asparagine (Asn), γ-aminobutyric acid (GABA), and glutamine (Gln), each with a purity ≥99%, were obtained from Macklin Biochemical Technology Co., Ltd. (Shanghai, China).

6-Aminoquinolyl-N-hydroxysuccinimidyl carbamate (AQC, purity 97%) and anhydrous sodium tetraborate (purity 99.5%) were purchased from Yuanye Biotechnology Co., Ltd. (Shanghai, China). Chromatographic-grade reagents, including anhydrous disodium hydrogen phosphate, anhydrous potassium dihydrogen phosphate, triethylamine hydrochloride, and phosphoric acid, were sourced from Aladdin Biochemical Technology Co., Ltd. (Shanghai, China). HPLC-grade acetonitrile was purchased from MREDA Technology Co., Ltd. (Beijing, China).

### 2.3. Quantification of Free Amino Acids

FAAs were quantified following previously reported procedures [18,19], with minor modifications as described below.

#### 2.3.1. Sample Preparation

Frozen litchi arils were ground into fine powder under liquid nitrogen. An aliquot of the powder (0.5 g) was weighed into a 10 mL centrifuge tube, followed by the addition of 8 mL of hot water. The mixture was extracted using ultrasonic-assisted heating for 10 min. After cooling to room temperature, the extract was centrifuged at 13,000 rpm for 5 min. The resulting supernatant was filtered through a 0.22 µm hydrophilic membrane, diluted threefold with ultrapure water, and subjected to derivatization.

#### 2.3.2. Derivatization Procedure

A 10 μL aliquot of either sample or standard solution was transferred into a 1.5 mL centrifuge tube, followed by the addition of 70 μL borate buffer and 20 µL of AQC derivatization reagent. The mixture was vortexed for 10 s, allowed to stand at room temperature for 2 min, and then incubated at 55 °C for 10 min. The resulting derivatized solution was transferred to an HPLC for analysis.

#### 2.3.3. Mobile Phase Preparation

The stock solutions were prepared as follows: Stock Solution 1, 800 mM Na_2_HPO_4_ (pH 8.90); Stock Solution 2, 800 mM KH_2_PO_4_ (pH 4.28); and Stock Solution 3, 1 M Triethylamine Hydrochloride.

Eluent A was prepared by mixing 5 mL of Stock Solution 1, 95 mL of Stock Solution 2, 10 mL of Stock Solution 3, and 54 mL of acetonitrile, with the final volume adjusted to 1000 mL with ultrapure water. Eluent B was prepared by mixing 100 mL of Stock Solution 2 and 500 mL of acetonitrile, with the final volume adjusted to 1000 mL with ultrapure water. Both eluents were filtered through a 0.45 µm membrane and degassed via ultrasonication before use.

#### 2.3.4. Chromatographic Conditions

HPLC analyses were performed on an Agilent 1260 Infinity system (Agilent Technologies, Santa Clara, CA, USA) equipped with a fluorescence detector (G7121A). Separation was achieved on a Poroshell 120 CS-C18 column (4.6 × 150 mm, 2.7 µm) maintained at 55 °C. The injection volume was 5 μL. Detection was conducted using fluorescence excitation and emission wavelengths of 250 nm and 395 nm, respectively. Elution was performed under a gradient program, as detailed in Supplementary Table S1.

### 2.4. Data Analysis

All experimental data were processed using Microsoft Excel 2019 and expressed as the mean ± standard deviation (SD) of three independent replicates. Data were analyzed on a fresh-weight basis to represent the natural physiological composition of the fruit at harvest. Nonparametric tests and correlation analyses were performed using SPSS 20.0 (IBM, Armonk, NY, USA). For variables showing significant differences in the Kruskal–Wallis test (*p* < 0.05), post hoc pairwise comparisons were conducted using Dunn’s test with Bonferroni correction to adjust for multiple comparisons. Statistical graphs were generated using GraphPad Prism 9.0 (GraphPad Software, San Diego, CA, USA). Based on the quantified concentrations of 20 free amino acids across different litchi varieties, a clustered heatmap was generated using TBtools-II (version 2.336). All figures were subsequently refined for publication using Inkscape software (version 1.2).

## 3. Results and Discussion

### 3.1. Variation in Free Amino Acid Content Across 148 Litchi Germplasm Accessions

**Figure 1:**
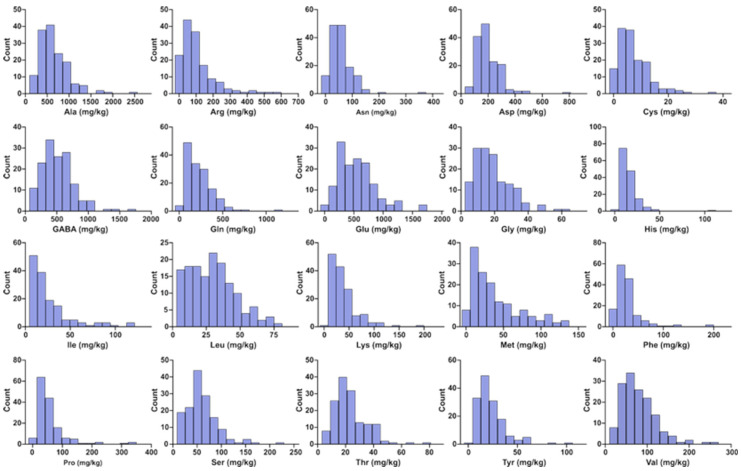
Frequency distribution of the FAA content across 148 litchi germplasm accessions. The histogram was constructed using the mean FAA value determined for each accession.

The FAA profiles in the aril of 148 litchi germplasm accessions were analyzed utilizing HPLC. Twenty distinct free amino acids were identified and quantified, including nineteen proteinogenic amino acids (excluding tryptophan) and one nonproteinogenic amino acid, GABA. All target compounds were effectively separated under the established chromatographic conditions (Supplementary Figure S1), and concentrations are reported on a fresh-weight basis (mg/kg) for consistency. The frequency distribution of the twenty FAAs across the 148 litchi accessions is shown in Figure 1. The total free amino acid (TFAA) content spanned from 965.34 mg/kg in ‘Anliang’ to 6636.28 mg/kg in ‘Tianshuili’, with a mean value of 2791.50 mg/kg and a coefficient of variation (CV) of 34.04% (Table 1). This wide range indicates substantial variation in FAA accumulation among litchi germplasms. This concentration range is comparable to that reported for peach pulp (2981.10 ± 1500.94 mg/kg) but generally lower than that in *Actinidia arguta* (3842.0–25,905.6 mg/kg) [20,21].

Among the FAAs detected in litchi fruit, Ala exhibited the highest mean concentration at 662.85 mg/kg (Table 1). This finding is consistent with previous reports on five elite and nine major litchi cultivars [18,22]. Its content ranged from 161.22 mg/kg (‘Baisha Hemaoli No. 2’) to 2497.77 mg/kg (‘Tianshuili’), constituting 13.46% to 43.61% of the TFAA (Table 1; Figure 1). Glu was the second most abundant FAA, with a mean concentration of 553.22 mg/kg. Its levels ranged from 33.89 mg/kg (‘Zengcheng Jinfeng’) to 1699.27 mg/kg (‘Huangye’), representing 1.28–46.68% of the TFAA (Table 1; Figure 1). GABA ranked third, displaying a mean concentration of 523.10 mg/kg. The concentration of GABA ranged from 87.13 mg/kg (‘Zengcheng Jingfeng’) to 1703.36 mg/kg (‘Dalongli’), comprising 7.42% to 40.80% of the TFAA (Table 1; Figure 1).

The dominance of Ala, Glu, and GABA is noteworthy from both metabolic and flavor perspectives. Glutamate serves as a central metabolite in plant carbon and nitrogen metabolism, functioning both as a protein constituent and as the direct biosynthetic precursor of GABA [23]. GABA, a bioactive compound found in fruits, plays a critical role in plant responses to abiotic stress [24]. In mammals, GABA serves as a major inhibitory neurotransmitter and exhibits physiological functions such as antihypertensive and anxiolytic effects, immune enhancement, and renal function improvement [25]. Notably, previous studies have demonstrated that GABA from litchi can effectively modulate gut microbiota dysbiosis in mice, thereby alleviating weight gain, fat accumulation, and oxidative damage, while also improving serum lipid profiles and liver function, indicating its potential anti-obesity effects [26].

In litchi fruit, Gln was the fourth most abundant FAA, with a mean concentration of 229.67 mg/kg (Table 1). Its content varied from 37.30 mg/kg in ‘Dadongli’ to 1126.07 mg/kg in ‘Guinuo’, representing 1.65% to 27.16% of the TFAA (Supplementary Tables S2 and S3). In plants, Gln not only serves as a major source of glutamate in metabolism but also functions as a signaling molecule involved in regulating plant metabolism, somatic embryogenesis, shoot organogenesis, and stress or defense responses [27]. Recent studies suggest that localized secretion of Gln by plant roots can precisely modulate the spatial distribution of rhizosphere microbiota [28].

Asp was the fifth most abundant FAA in litchi, with a mean concentration of 202.98 mg/kg (Table 1). Its content ranged from 64.05 mg/kg in ‘Yeshenglizhi 9 Hao’ to 768.39 mg/kg in ‘Zili’, constituting 2.45% to 25.71% of the TFAA (Supplementary Tables S2 and S3). As a metabolic product of glutamate, aspartate acts as a common precursor for the biosynthesis of several essential amino acids, including lysine, threonine, methionine, and isoleucine [29]. Consequently, its metabolic pathways are a major focus of research on amino acid biosynthesis in crops such as rice [29,30]. The variation in Asp content observed here may reflect differences in the flux through these branching biosynthetic pathways among different litchi genotypes.

The remaining free amino acids (Arg, Asn, Cys, Gly, His, Ile, Leu, Lys, Met, Phe, Pro, Ser, Thr, Tyr, and Val) each had a mean concentration constituting less than 5% of the TFAA (Table 1). Despite their lower abundance, their quantitative variation reflects the diversity within the germplasm resources (Figure 1). Notably, the high coefficients of variation (CV > 90%) for Arg, Ile, Phe and Pro indicate substantial genetic diversity for these components, presenting opportunities for breeding programs aimed at modifying specific amino acid profiles. Detailed FAA data are provided in Supplementary Tables S2 and S3.

### 3.2. Correlation Analysis of Free Amino Acid Contents

**Figure 2:**
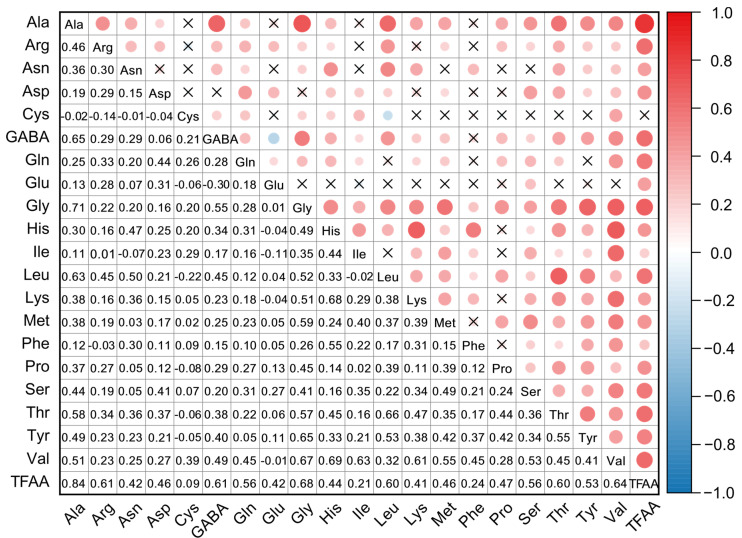
Correlation heatmap of 20 individual FAAs and the TFAA content across 148 litchi germplasm accessions. The color scale indicates the Pearson correlation coefficient (r), with red representing positive correlations and blue representing negative correlations. Cells marked with “×” denote non-significant correlations (*p* > 0.05).

A Pearson correlation analysis was performed to elucidate the relationships among individual components and with the TFAA content (Figure 2). The results revealed that TFAA content was significantly and positively correlated with most amino acids. The strongest correlation was observed with Ala(*r* = 0.84), followed by Gly (*r* = 0.68) and Val (*r* = 0.64). This finding confirms that Ala is the predominant contributor to the TFAA pool in litchi aril.

Most amino acids exhibited positive correlations of varying strength (*r* > 0.3), suggesting coordinated regulation of their biosynthetic pathways. For example, strong positive correlations were observed between Ala and Gly (*r* = 0.71) and between Gly and Val (*r* = 0.67). A noteworthy negative correlation (*r* = −0.30) was observed between GABA and its direct biosynthetic precursor, Glu. This inverse relationship suggests active metabolic conversion in litchi fruit, whereby Glu is enzymatically converted into GABA through glutamate decarboxylase (GAD) activity [31]. Supporting this notion, studies in longan have demonstrated that *DlGAD3* promotes GABA accumulation and contributes to the variation in GABA content between cultivars BS1 and CX [32]. This reciprocal pattern aligns with observations in other fruit species such as tomato [33], reinforcing the view that the GABA biosynthetic pathway acts as a key metabolic node modulating the relative abundance of these two amino acids in litchi.

### 3.3. Nutritional and Taste Profile Evaluation of Amino Acids in Litchi Germplasm Accessions

Essential amino acids (EAAs), which cannot be synthesized by the human body and must be obtained through the diet, include Lys, Thr, Met, Trp, Phe, Ile, Leu, and Val [30]. Consistent with most fruits, which are generally not major sources of protein and EAAs, litchi arils exhibited modest EAA levels. Among the 148 litchi cultivars analyzed, the mean EAA content was 266.91 mg/kg, ranging from 86.79 mg/kg in ‘Renshen 2’ to 812.34 mg/kg in ‘Wujun 3’ (Supplementary Table S2). The World Health Organization/Food and Agriculture Organization (WHO/FAO) recommends an ideal protein pattern where the essential-to-total amino acid ratio (E/T ratio) is approximately 40% [34]. However, none of the litchi accessions met this standard; the highest EAA/TAA ratio was only 20.77%, observed in the ‘Chitangli’ cultivar (Supplementary Table S3). This result reinforces that litchi, like other fresh fruits, should not be considered a primary source of dietary protein or EAAs, but rather valued for its other nutritional and sensory attributes.

Beyond their nutritional roles, FAAs are crucial contributors to food flavor. Based on their taste profiles, the FAAs in litchi arils were categorized as umami (UAA: Asp, Glu), sweet (SAA: Ala, Gly, Lys, Pro, Ser, Thr), or bitter (BAA: Arg, His, Ile, Leu, Met, Phe, Tyr, Val) [35,36]. The mean contents of these taste-active categories followed the order: SAA > UAA > BAA (Supplementary Table S2). Specifically, SAAs were the most abundant group, with a mean content of 863.27 mg/kg (30.19% of TFAA), ranging from 220.22 (‘Baisha Hemaoli 2Hao’) to 2901.05 mg/kg (‘Tianshuili’). UAAs followed, with a mean content of 756.20 mg/kg (28.02% of TFAA) and a range of 132.81 (‘Yeshenglizhi9Hao’) to 2028.10 mg/kg (‘Babiaoxiang’). BAAs were the least abundant, with a mean content of 352.97 mg/kg (12.35% of TFAA), varying between 81.17 (‘Renshen 2Hao’) and 873.59 mg/kg (‘Huagangguinuo’).

Because different amino acids have distinct taste thresholds, concentration alone cannot accurately reflect their contribution to flavor. Therefore, the taste activity value (TAV) was applied for further evaluation [36]. TAV is defined as the ratio of the concentration of a given taste compound to its corresponding taste threshold, and it reflects the relative contribution of that compound to the overall taste profile. A TAV greater than 1 indicates that the compound has a perceptible influence on taste, whereas a TAV less than 1 suggests a negligible direct contribution [37]. The analysis revealed that among the 20 FAAs, only Glu (umami) and Ala (sweet) had mean TAVs greater than 1, identifying them as the primary taste-active compounds in litchi (Table 2). Across the cultivars, the TAV of Glu ranged from 0.11 (‘Zengcheng Jinfeng’) to 5.66 (‘Huangye’), with a mean of 1.84, establishing it as the primary determinant of umami (Supplementary Table S4). The TAV of Ala ranged from 0.27 (‘Anliang’) to 4.16 (‘Tianshuili’), with a mean of 1.10, indicating its role in sweetness. Among bitter amino acids, although the mean TAV for Arg was only 0.22, it exceeded the taste threshold in cultivars such as ‘Huagang Guinuo’ (1.13) and ‘Babiaoxiang’ (1.07). It is important to note that amino acids with TAV <1 can still modulate flavor through synergistic, additive, or suppressive interactions [38]. For instance, sub-threshold bitter amino acids (e.g., Phe, Tyr) can enhance umami and sweet tastes [39]. However, because no sensory evaluation was conducted in the present study, the relationship between TAV-based predictions and actual flavor perception remains inferential. Future work integrating sensory validation and metabolomic–sensory correlation analysis will be essential to confirm how variation in FAA composition translates to perceived flavor differences among litchi cultivars.

### 3.4. Variation in Free Amino Acid Profiles Across Different Types

**Figure 3:**
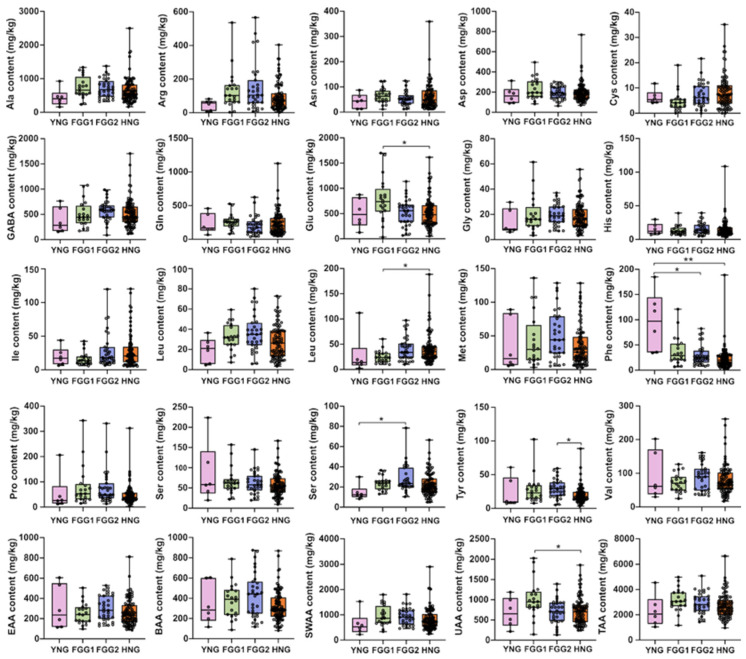
Range and distribution of FAA contents among four genetic groups of litchi germplasm. The horizontal line within each box represents the median value. Significant differences between groups (*p* < 0.05), as determined by the Kruskal–Wallis test, are indicated by different asterisks (* for *p* < 0.05; ** for *p* < 0.01). Non-significant pairs are not marked.

Previous research indicates that wild litchi populations in Yunnan and Hainan underwent independent domestication, giving rise to very early maturing and late-maturing cultivars, respectively. Subsequent hybridization between these two lineages is thought to have produced the early and mid-maturing cultivars [1]. In line with this domestication history, the 148 litchi accessions in this study were categorized into four established genetic groups: the Yunnan Group (YNG), the Hainan Group (HNG), and two hybrid groups (FGG1 and FGG2), to compare their FAA profiles [5]. Comparative analysis revealed distinct FAA profiles among these groups (Figure 3). Specifically, the YNG was characterized by significantly higher concentrations of Phe than the FGG2 and HNG groups, and higher Thr than the FGG2 group. The FGG1 group exhibited significantly higher concentrations of Glu and Lys than the HNG group, resulting in a higher overall content of flavor-active amino acids. Additionally, the FGG2 group showed a significantly higher Tyr content than the HNG group (Figure 3).

Litchi cultivars can also be classified into three sugar-accumulation types based on the ratio of hexoses to sucrose in the mature aril: hexose-dominant (ratio > 2), sucrose-dominant (ratio < 1), and intermediate (ratio 1–2) [40,41]. To investigate the potential link between sugar and amino acid metabolism, we compared the FAA profiles across these three types. Our analysis showed no significant differences for most FAAs among the three sugar-accumulation types. The only exceptions were Ser and Met, which were found at significantly higher levels in the hexose-dominant type compared to the intermediate type (Supplementary Figure S2). These results suggest that there is no broad association between the primary sugar accumulation pattern and the FAA profile in mature litchi arils.

### 3.5. Hierarchical Cluster Analysis of 148 Litchi Germplasm Accessions Based on Free Amino Acid Composition

Cluster analysis is an effective methodology for classifying germplasm and investigating genetic diversity, facilitating the targeted selection of cultivars for specific breeding objectives [20,42]. Recent metabolomic studies have identified amino acid metabolism and phenylalanine metabolism as key pathways for differentiating litchi cultivars and developmental stages [43]. To elucidate the FAA profiles among the 148 litchi germplasms, hierarchical cluster analysis was performed using heatmap visualization based on the quantitative data of 20 FAA. As shown in Supplementary Figure S3, the germplasms were grouped into four distinct clusters. Group I was characterized by a significantly elevated GABA content. Group II showed notably high Ala levels, whereas Group III was characterized by high Glu concentrations. Group IV, which included only five cultivars, displayed generally low levels of all detected amino acids. Concurrently, the 20 amino acids were broadly divided into two main clusters, one of which predominantly included GABA, Ala, and Glu (Supplementary Figure S3).

**Figure 4:**
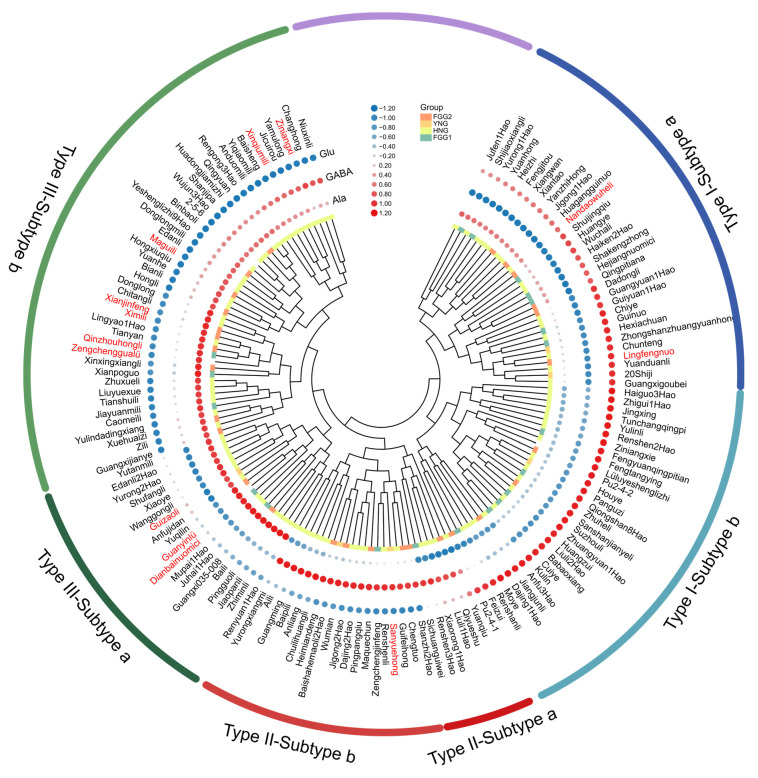
Hierarchical clustering and heatmap visualization of GABA, Glu, and Ala contents across 148 litchi germplasm accessions. Each row represents a litchi accession, and each circle corresponds to one amino acid. Color intensity indicates the relative concentration of each amino acid (blue = low; red = high). Cultivar names highlighted in red denote widely cultivated or newly released elite varieties.

Given the quantitative importance of GABA, Glu, and Ala in litchi fruit and their strong influence on flavor quality and functional properties, an additional cluster analysis was conducted focusing specifically on these three amino acids. The results revealed a clear classification into three primary types (Figure 4). Type I was defined as the Glu-accumulating type and was further divided into two subtypes: Subtype I-a, containing 31 accessions characterized by co-accumulation of Glu and Ala, and Subtype I-b, comprising 30 accessions in which Glu was the predominantly accumulated amino acid. Type II was identified as the GABA-accumulating type, also divided into two subtypes: Subtype II-a, comprising six accessions that accumulated both GABA and Glu, and Subtype II-b, containing 19 accessions in which GABA was the predominant accumulated component. Type III was characterized as the Ala-accumulating type, comprising two subtypes: Subtype III-a, which included 19 accessions primarily accumulating Ala, and Subtype III-b, containing 43 accessions characterized by co-accumulation of Ala and GABA. This hierarchical classification provides a robust framework for the exploitation of distinctive litchi germplasm and establishes a foundational basis for targeted breeding strategies. The emergence of these chemotypes likely reflects fundamental differences in central carbon–nitrogen metabolic fluxes. The Glu-accumulating type may be associated with enhanced nitrogen assimilation and processing, potentially driven by coordinated activity of glutamine synthetase (GS) and glutamate synthase (GOGAT) [44]. In contrast, the GABA-accumulating type suggests a metabolic redirection in which glutamate is preferentially channeled into the GABA shunt, a conversion catalyzed by GAD [32]. The Ala-accumulating type, meanwhile, likely indicates a metabolic state characterized by elevated glycolytic flux, generating pyruvate that is subsequently converted into alanine through alanine aminotransferase [45].

Interestingly, these chemotypes also show partial correspondence with litchi population structure. Glu-rich accessions occurred more frequently in the YNG and early maturing FGG1 groups, whereas FGG2 and the HNG group contained a higher proportion of both Ala-dominant and Glu-dominant chemotypes (Supplementary Figure S4). This pattern is consistent with previous findings that the Yunnan and Hainan lineages exhibit a substantial admixture in the Guangxi–Guangdong border region—the proposed domestication center of litchi—followed by dispersal into Guangdong, Fujian, Southeast Asia, and beyond [5]. Together, these findings suggest that the three chemotypes arise from an interplay between metabolic specialization and population-specific genetic differentiation. Future work integrating enzyme activity assays, transcriptomic profiling, and genome-wide association studies (GWAS) will be essential to identify the specific loci regulating Glu, GABA, and Ala accumulation and clarify how population history and selection have shaped amino acid variation in litchi.

## 4. Conclusions

This study provides a comprehensive analysis of twenty free amino acids across 148 litchi germplasm accessions. The results definitively identified Ala, Glu, and GABA as the predominant free amino acids, establishing them as key determinants of the fruit’s sweetness, umami taste, and functional properties. A wide variation in the total free amino acid content was observed, exemplified by the highest levels in ‘Tianshuili’ and the lowest in ‘Anliang’. Furthermore, significant differences in specific FAA profiles were linked to the genetic background of the accessions. Cluster analysis based on the three dominant amino acids classified the germplasm into three distinct chemotypes: Glu-accumulating, GABA-accumulating, and Ala-accumulating, providing a clear strategy for cultivar selection and breeding. In summary, this study presents the first large-scale, systematic assessment of free amino acid composition in litchi germplasm. The findings establish a scientific basis for selecting cultivars with desirable amino acid traits and lay a foundation for identifying candidate genes involved in amino acid metabolism.

## Figures and Tables

**Table 1:** Statistical summary of FAA contents in 148 litchi germplasm accessions.

Amino Acids	Mean (mg/kg)	SD (mg/kg)	Median (mg/kg)	Min (mg/kg)	Max (mg/kg)	CV (%)
**Ala**	**659.35**	**351.60**	**566.80**	**141.62**	**2497.77**	**53.33**
Arg	109.03	103.93	77.54	2.55	566.06	95.32
Asn	59.59	43.34	51.21	11.15	359.16	72.73
Asp	202.46	91.81	188.64	64.05	768.39	45.35
Cys	7.40	5.87	6.06	<LOD ^1^	35.19	79.27
**GABA**	**520.47**	**262.86**	**493.76**	**87.13**	**1703.36**	**50.50**
Gln	228.20	158.03	202.38	9.89	1126.07	69.25
**Glu**	**551.16**	**321.47**	**523.97**	**33.89**	**1699.27**	**58.33**
Gly	18.93	10.68	16.65	3.06	61.42	56.41
His	15.28	11.18	12.85	1.56	108.60	73.14
Ile	26.02	23.58	18.45	3.65	120.48	90.64
Leu	29.46	16.92	27.51	3.30	80.05	57.43
Lys	38.15	27.61	31.43	2.45	188.42	72.38
Met	38.75	33.05	29.27	2.65	136.06	85.29
Phe	29.35	28.57	22.61	3.59	188.63	97.34
Pro	58.50	54.48	43.21	2.68	342.77	93.13
Ser	60.54	32.39	55.11	9.18	223.93	53.50
Thr	23.48	11.58	20.99	5.06	78.43	49.33
Tyr	22.90	14.97	19.49	3.19	102.25	65.37
Val	80.26	42.67	72.76	16.77	261.17	53.16
TFAA	2778.45	960.22	2693.42	847.11	6636.28	34.56

^1^ <LOD indicates below the limit of detection (0.0086 mg/kg) [18].

**Table 2:** Statistical summary of TAVs of FAAs in lychee aril.

Taste	FAA	Mean	SD	Median	Min	Max
umami	Asp	0.20	0.09	0.19	0.06	0.77
umami	Glu	1.84	1.07	1.76	0.11	5.66
sweet	Ala	1.10	0.58	0.95	0.27	4.16
sweet	Gly	0.01	0.01	0.01	0.00	0.05
sweet	Ser	0.04	0.02	0.04	0.01	0.15
sweet	Thr	0.01	0.00	0.01	0.01	0.03
sweet/bitter	Lys	0.08	0.06	0.06	0.00	0.38
sweet/bitter	Pro	0.02	0.02	0.01	0.00	0.11
bitter	Arg	0.22	0.21	0.16	0.01	1.13
bitter	His	0.08	0.06	0.06	0.02	0.54
bitter	Ile	0.03	0.03	0.02	0.01	0.13
bitter	Leu	0.02	0.01	0.01	0.00	0.04
bitter	Met	0.13	0.11	0.10	0.01	0.45
bitter	Phe	0.03	0.03	0.03	0.00	0.21
bitter	Tyr	0.00	0.00	0.00	0.00	0.01
bitter	Val	0.20	0.11	0.18	0.04	0.65

## Data Availability

The original contributions presented in this study are included in the article/Supplementary Material. Further inquiries can be directed to the corresponding author.

## Supplementary Materials

The following supporting information can be downloaded at: https://www.mdpi.com/article/10.3390/foods14234051/s1, Figure S1: HPLC chromatograms of the 20 amino acids; Figure S2: Range and distribution of free amino acid contents among three sugar-accumulation types of litchi germplasm; Figure S3: Hierarchical clustering and heatmap visualization of 20 FAA contents across 148 litchi germplasm accessions; Figure S4: Distribution of the three amino acid chemotypes across four litchi genomic groups; Table S1: Gradient elution program; Table S2: The mean content of free amino acids in 148 litchi germplasm accessions; Table S3: The relative content of free amino acids in 148 litchi germplasm accessions; Table S4: Taste activity values of flavor amino acids in 148 litchi germplasm accessions.
