# Supplementary material for: Comprehensive Profiling of Free Amino Acids in Litchi (Litchi chinensis Sonn.) Germplasm and Their Implications for Flavor Quality
Source: Foods. 2025 Nov 26;14(23):4051. doi: 10.3390/foods14234051 (PMC12691768; doi:10.3390/foods14234051)
Supplement: Supplementary file 1 [file foods-14-04051-s001.zip › Supplementary Figures.pdf]

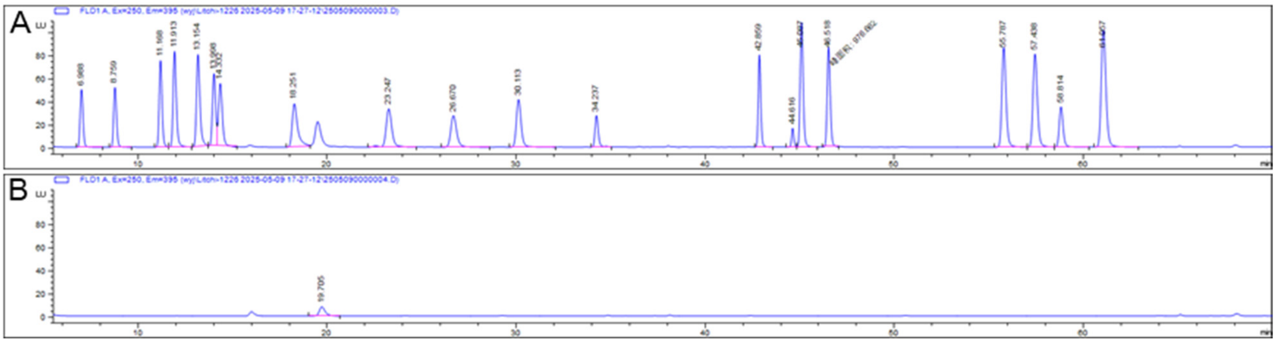

Figure S1. HPLC chromatograms of the 20 amino acids. (A) Standard sample, (B) Blank sample. The 20 amino acids are, in order of elution: Asp (6.988 min); Glu (8.759 min); Asn (11.168 min); Ser (11.913 min); His (13.154 min); Gln (13.998 min); Gly (14.332 min); Arg (18.251 min); Thr (23.247 min); Ala (26.670 min); GABA (30.113 min); Pro (34.237 min); Tyr (42.859 min); Cys (44.616 min); Val (45.087 min); Met (46.518 min); Ile (55.787 min); Leu (57.438 min); Lys (58.814 min); Phe (61.057 min).

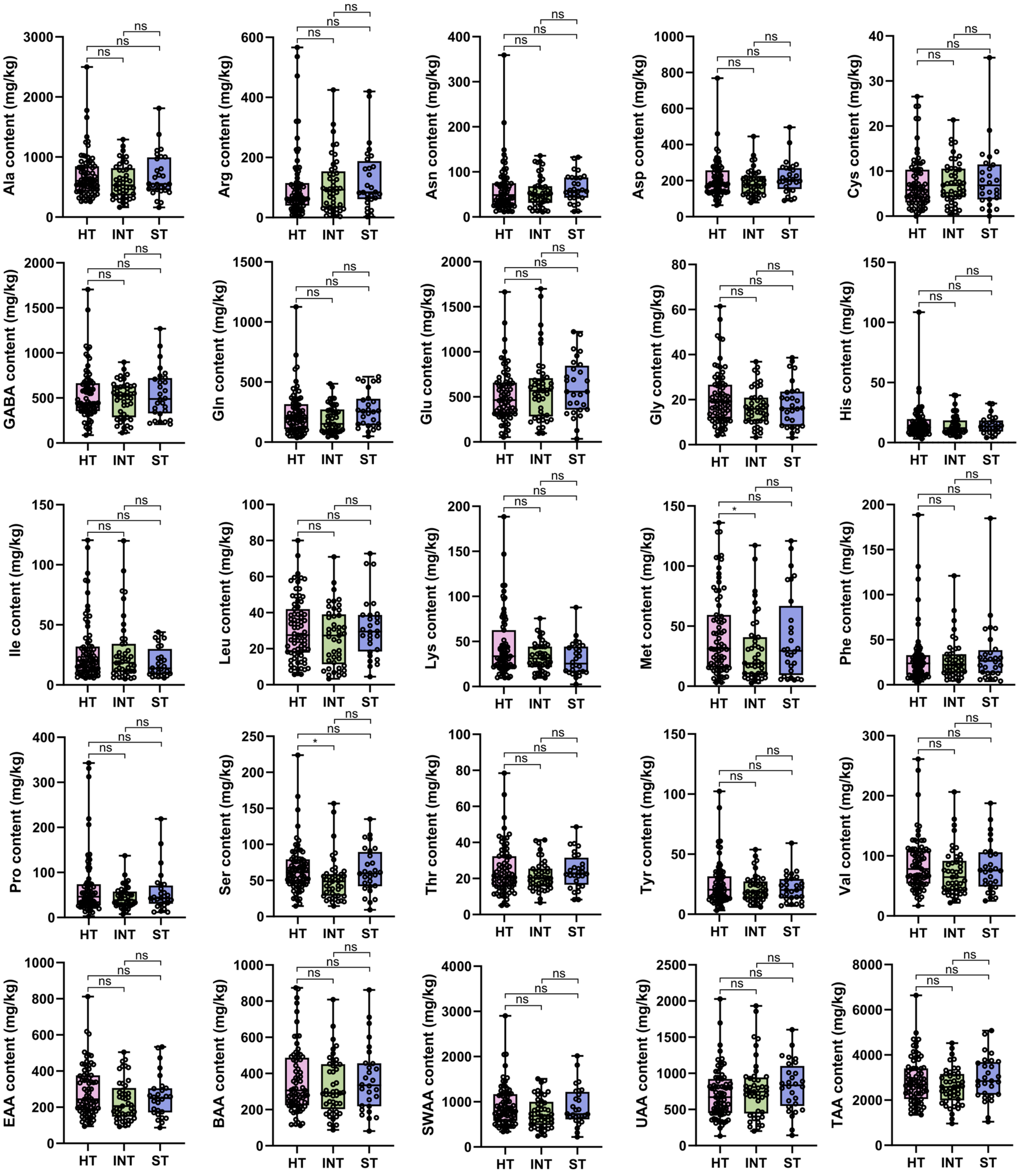

Figure S2. Range and distribution of free amino acid contents among three sugar-accumulation types of litchi germplasm. The horizontal line within each box represents the median value. *P* values were determined using the Kruskal–Wallis test ($P < 0.05$).

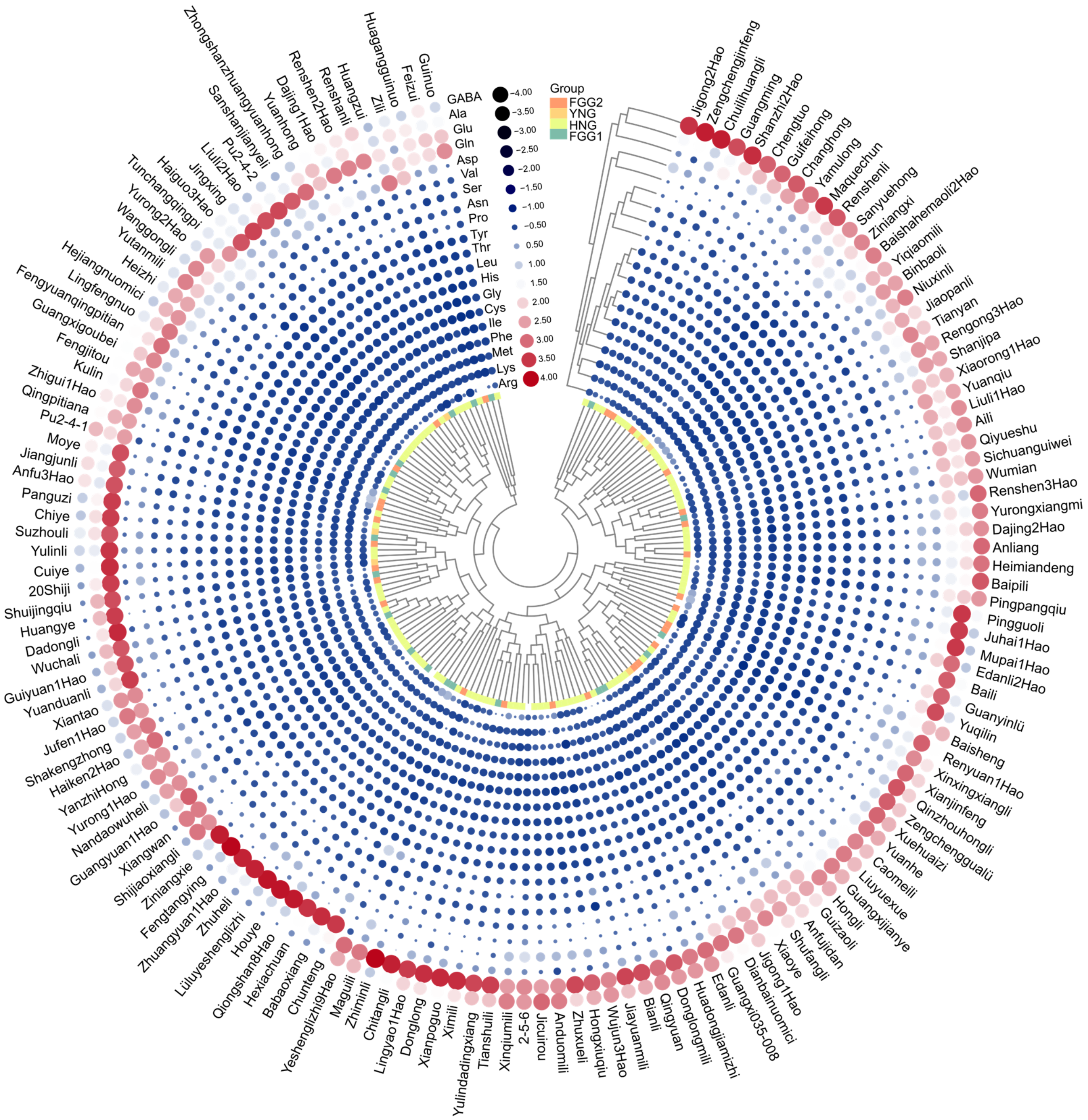

Figure S3. Hierarchical clustering and heatmap visualization of 20 FAA contents across 148 litchi germplasm accessions. Each row represents a litchi accession, and each circle corresponds to an FAA. Color intensity indicates the relative concentration of each FAA (blue = low; red = high).

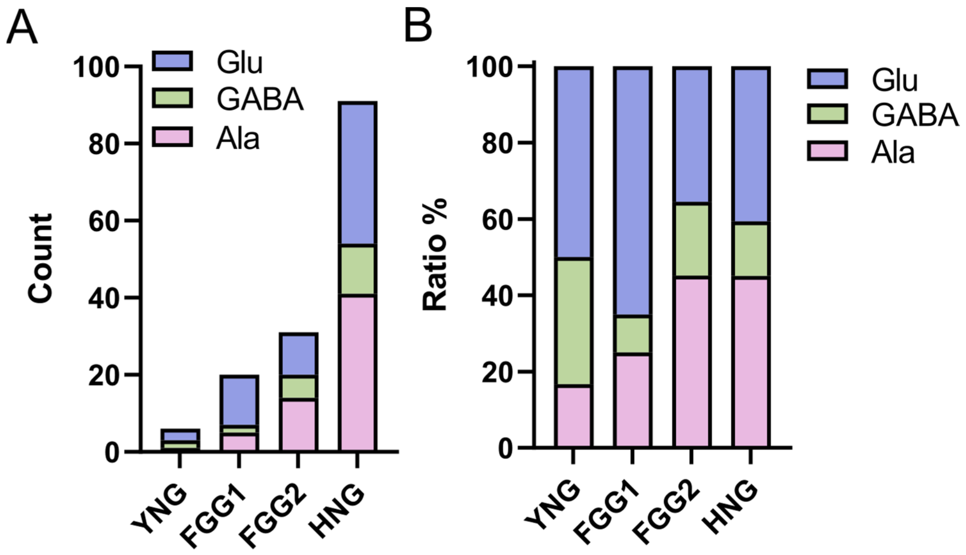

Figure S4. Distribution of the three amino acid chemotypes across four litchi genomic groups. Stacked bar plots illustrate the absolute number (A) and relative proportion (B) of Glu- (blue), GABA- (green), and Ala-accumulating (red) accessions within the YNG, FGG1, FGG2, and HNG groups.
